# Assessment of methods and analysis of outcomes for comprehensive optimization of nucleofection

**DOI:** 10.1186/1479-0556-7-6

**Published:** 2009-05-11

**Authors:** Christopher Bradburne, Kelly Robertson, Dzung Thach

**Affiliations:** 1 Center for Bio/Molecular Science and Engineering, US Naval Research Laboratory, Code 6900, 4555 Overlook Ave SW, Washington DC 20375, USA; 2 NIAID, NIH, DHHS, Bldg 33, Room 3W10A.6, 33 North Drive, MSC 3203 Bethesda, Maryland 20892-3203, USA

## Abstract

**Background:**

Nucleofection is an emerging technology for delivery of nucleic acids into both the cytoplasm and nucleus of eukaryotic cells with high efficiency. This makes it an ideal technology for gene delivery and siRNA applications. A 96-well format has recently been made available for high-throughput nucleofection, however conditions must be optimized for delivery into each specific cell type. Screening each 96-well plate can be expensive, and descriptions of methods and outcomes to determine the best conditions are lacking in the literature. Here we employ simple methods, including cell counting, microscopy, viability and cytotoxicity assays to describe the minimal experimental methods required to optimize nucleofection conditions for a given cell line.

**Methods:**

We comprehensively measured and analyzed the outcomes of the 96-well nucleofection of pmaxGFP plasmids encoding green fluorescent protein (GFP) into the A-549 human lung epithelial cell line. Fluorescent microscopy and a plate reader were used to respectively observe and quantify green fluorescence in both whole and lysed cells. Cell viability was determined by direct counting/permeability assays, and by both absorbance and fluorescence-based plate reader cytotoxicity assays. Finally, an optimal nucleofection condition was used to deliver siRNA and gene specific knock-down was demonstrated.

**Results:**

GFP fluorescence among conditions ranged from non-existent to bright, based upon the fluorescent microscopy and plate reader results. Correlation between direct counting of cells and plate-based cytotoxicity assays were from R = .81 to R = .88, depending on the assay. Correlation between the GFP fluorescence of lysed and unlysed cells was high, ranging from R = .91 to R = .97. Finally, delivery of a pooled sample of siRNAs targeting the gene relA using an optimized nucleofection condition resulted in a 70–95% knock down of the gene over 48 h with 90–97% cell viability.

**Conclusion:**

Our results show the optimal 96-well nucleofection conditions for the widely-used human cell line, A-549. We describe simple, effective methods for determining optimal conditions with high confidence, providing a useful road map for other laboratories planning optimization of specific cell lines or primary cells. Our analysis of outcomes suggests the need to only measure unlysed, whole-cell fluorescence and cell metabolic activity using a plate reader cytotoxicity assay to determine the best conditions for 96-well nucleofection.

## Background

The transfection of molecules into mammalian cells is an essential tool for the study of gene function, the delivery of genetic therapy agents, and for cell diagnostics and imaging. Many different transfection methods have been developed, including chemical (reviewed in [1]), 'biolistic' or ballistic bombardment [2], viral [3], electroporation [4,5], microinjection, and liposomal[6] delivery technologies. Most of these approaches are limited by low transfection efficiencies, high cytotoxicity, and the inability to deliver nucleic acid past the nuclear barrier within the cell, especially in primary cell lines.

Nucleofection is an emerging technology for intracellular molecular delivery. It is typically used in a single-cuvette format for delivery of nucleic acids such as plasmids and siRNA, and has been successfully used to deliver nucleic acids into human embryonic stem cells, adult stem cells, myoblasts, monocytes, human keratinocytes, murine stem cells, and many others [7-14]. A non-viral delivery system, it has shown promise in the successful transfection of normally hard to transfect cells [9], however, it could also potentially be used to deliver proteins, inorganic compounds, nanoparticles, drugs, and toxins. Characteristic of nucleofection is its ability to deliver molecules into the nucleus as well as the cytoplasm, which offers a distinct advantage over non-viral delivery strategies that generally only deliver into the cytoplasm [15,16].

Nucleofection is achieved by combining low voltage electroporation with one of several reagents to allow the efficient transfer of nucleic acids into cells while minimizing toxicity [17,18]. The reagents are proprietary in nature, but generally consist of a combination of modular protein complexes that combine with charged particles such as nucleic acids, forming a nucleoprotein complex [19]. Different protein complexes facilitate separate functions, such as cell membrane association, translocation, endosomal release, and nuclear transport [19]. The entire procedure has been optimized in the single-cuvette format for a variety of mammalian cell types, and recently for a 96-well shuttle system. However, the shuttle system must be optimized for each cell type, which involves the screening of up to 96 conditions to select the best one for efficient nucleofection. The parametric conditions are a combination of three proprietary reagents and 31 different electrical pulse-shaping options. The reagents are expensive, costing several hundred dollars per plate, while descriptions of the methods/outcomes for the 96 conditions and easy-to-use protocols for the evaluation of the results are lacking. We therefore performed several simple, duplicate assays, and then compared their outcomes to determine the simplest, most cost-effective requirements to optimize any given cell line. The techniques we evaluate here include fluorescence microscopy, a fluorescence plate reader, cell permeability assays/direct cell counting, and both absorbance- and fluorescence-based cytotoxicity assays. In addition, we specifically discuss the optimal nucleofection conditions for a human epithelial cell line: A549, and recommend the minimal assays needed for evaluating optimal delivery conditions and delivery outcomes using this shuttle system. The results described here will serve as a useful reference for others wanting to optimize the 96-well shuttle system for any cell line.

## Methods

### Initial Nucleofection Optimization

Nucleofection was carried out using the Cell Line Optimization 96-well Nucleofector Kit from Amaxa  according to the manufacturer's recommendations. Briefly, A549 cells (ATCC – Manassas, VA) were grown to 85% confluency in complete media (Dulbecco's Modified Eagle's Medium (DMEM) (Cellgro-Herndon, Virginia), supplemented with 10% (v/v) fetal calf serum (HyClone – Logan, Utah), 1% (v/v) penicillin, 1% (v/v) streptomycin (Sigma – St. Louis, Missouri)) and detached from culture flasks using trypsin (Cellgro). Complete media was added and the cells were split into three aliquots each containing approximately 8.75 × 10^6 ^cells. The aliquots were centrifuged at 800 rpm for 10 minutes and the media was completely removed from the pellet. Each of the three cell pellets was re-suspended in the three different nucleofection solutions (SE, SF, and SG) and 12.8 μg pMAX GFP plasmid was added to each solution. Each well in the 96-well nucleofection plate was loaded with 20 μL of one of the three nucleofection solutions (approximately 275,000 cells) and the plate was loaded into the Amaxa 96-well Shuttle for nucleofection. Upon completion of the nucleofection program and after a 10 minute incubation period, 80 μL of pre-warmed complete media was added to each well of the 96-well Nucleocuvette plate giving 100 μL total volume in each well. For recovery plates, two identical 96-well, flat-bottom plates, and 1 clear-bottomed/opaque walled 96-well plate (Becton-Dickenson) were then prepared by adding 25 μL of each nucleofected well to 175 μL of pre-warmed media. The opaque walled plate was used for a subsequent fluorescent assay measuring cell metabolic activity, in order to prevent any interference of fluorescence from neighbouring wells. In this way, each nucleofection condition had three identical growing conditions in the recovery plates. The cells were then incubated for 24–48 h in a humidified 37°C/5% CO_2 _atmosphere, and then used for either microscopy + fluorescence plate reading, absorbance or fluorescence cytotoxicity assay, or cell counting/Trypan Blue viability assay.

### Secondary Nucleofection Optimization

A second nucleofection optimization was performed using SE reagent, which allowed the further evaluation of this reagent in triplicate under all 32 nucleofection conditions. This optimization was performed as described above, but with the SE reagent substituted for the SF and SG reagents.

### Microscopy and GFP fluorescence detection

After 24 hrs, the cells in the clear-bottom, opaque-walled recovery plate were analyzed by both bright-field and fluorescence microscopy with a 20× objective using an Olympus microscope. For the GFP excitation, an Argon laser was used with λ_exe _= 488 nm. After microscopy, quantitative measurement of GFP/well was performed in two ways: first by direct measurement of whole-cell GFP in each well, and secondly by lysis of cells to release GFP in order to yield a more homogeneous measurement. Cell lysis was induced by the addition of 5 uL of 0.2 N HCl, and the fluorescence measured immediately after lysis. The GFP fluorescence was measured each time with a Tecan plate reader using λ_exe _= 485, λ_em _= 525 nm.

### Cell Number and Viability Determination

The actual cell number and viability was determined using a standard Trypan-Blue membrane permeability assay, in which all cells from each well in one of the non-opaque walled recovery plates were counted on a hematocytometer. In order to account for dead and dying cells that may have become detached, plates were centrifuged for 10 minutes at 800 rpm and the media was removed. The plate was then washed once with 1× DPBS, trypsinized, and complete media was added. The live and dead cells were then stained with trypan blue, counted, and percent viability was calculated as the number of live cells/total number of cells × 100.

### Toxicity Assays

The initial nucleofection optimization was evaluated using only GFP fluorescence, microscopy, and absolute cell number. For further evaluation and to alleviate the need for classical cell counting, the viability and cytotoxicity of cells from the SE optimization were analyzed using two different commercially available kits. First, the Celltiter 96 Aqueous One Solution Cell Proliferation Assay (Promega) measures live cell metabolic activity (live cell absorbance assay). Briefly, the presence of live cells is measured colorimetrically by the reduction of a tetrazolium salt substrate into a formazan product. NADPH provides the reducing power to catalyze the formazan conversion, resulting in a linear relationship between the amount of formazan produced, and the number of cells present. The formazan product in this assay is soluble, and can be detected using simple absorbance. The second assay used in this assessment was the MultiTox-Fluor Multiplex Cytotoxicity Assay (Promega), which is intended to measure live/dead cells simultaneously (live cell fluorescence assay). In this assay, a reagent containing both the live and dead cell indicators is used. The live cell indicator consists of a proprietary peptide substrate conjugated to a glycyl-phenylalanyl-amino-fluorocumarin (GF-AFC), which is permeable to the cell membrane. Entry into the cell membrane results in peptide cleavage by live cell proteases, and detection at 505 nm via excitation at 400 nm. The dead cell indicator is likewise a cell impermeable peptide substrate conjugated to a bis-alanyl-alanyl-phenylalanyl-rhodamine 110 (bis-AAF-R110), whose spectrophotometric properties are activated upon peptide cleavage (excitation at 485 nm/emission at 520 nm) However, for our comparisons, we only evaluated the use of the live cell, GF-AFC assay so it could be correlated to direct cell counting and metabolic activity measured by MTS. For each assay, cells were nucleofected and then allowed to proliferate for 48 hours before addition of the tetrazolium salt (AqueousOne), or the Live (GF-AFC) reagent of the MultiTox assay. Cells were then assayed according to the manufacturer's specifications for each kit.

### Data Bioinformatics

To determine the optimal nucleofection conditions, we converted the raw data obtained from the secondary optimization to a standardized form, clustered the standardized data, and generated a heat map. The heat map allows many data sets to be clustered, visualized, and compared with each other in order to determine the best conditions. Briefly, each data point was first standardized by subtracting the mean of the data set from each data point and then dividing by the standard deviation of the data set. Standardized and raw data for the secondary optimization can be viewed in the supplementary file [see Additional file 1]. Heat maps were then generated using dChip2005 , which uses hierarchical clustering to compare and group the data sets that have the highest degree of similarity. For the correlations (Table 1), the 3 trials were averaged and the r values were obtained using linear regression.

**Table 1:** Data correlation statistics from the secondary nucleofection optimization

Correlations	R value
Total Live Cells 24 hr. vs. Absorbance Assay 24 hr	.81
Total Live Cells 24 hr. vs. Fluorescence Assay 24 hr	.88
Absorbance Assay 24 hr vs. Fluorescence Assay 24 hr	.79
GFP (non-lysed) 24 hr vs. GFP (lysed) 24 hr	.91
GFP (non-lysed) 48 hr vs. GFP (lysed) 48 hr	.97
Absorbance Assay 48 hr vs. Fluorescence Assay 48 hr	.56

### siRNA delivery and qPCR

ON-TARGETplus SMARTpool siRNA constructs were purchased from Dharmacon Inc (Lafayette, CO) for rel-A (L-003533-00-0005). The siRNA preparations were re-suspended in 1× siRNA buffer (20 mM KCl, 6 mM HEPES-pH 7.5, 0.2 mM MgCl_2_) to working concentrations of 20 μM, and then delivered at concentrations of 0 nM (only 1× siRNA buffer), 100 nM, 250 nM, or 500 nM concentrations in the 96-well format using the nucleofector shuttle system. From the conditions determined in the optimization described below, each well contained 2.75 × 10^5 ^cells in Amaxa cell line solution SE, using program code 96-DS:150, and the standard control option. Transfected cells were split into 4 plates for recovery, resulting in 7 × 10^4 ^cells/well. Cells were counted and collected at 24 and 48 h following siRNA delivery by re-suspension in Cells-to-Signal lysis buffer (Applied Biosystems/Ambion, Austin, Texas, USA), and then qPCR was performed using a lysate equivalent to 100 cells/qPCR reaction. Real-time, quantitative PCR (qPCR) was performed according to the manufacturer's specifications using Taqman primer/probe sets purchased from Applied Biosystems, Inc (Foster City, California, USA) for Rel A (Primer set ID: Hs00153294_m1).

## Results

### Optimization Strategy

To determine the optimal condition for nucleofection of the A-549 epithelial cell line, an initial optimization experiment was performed as described by the manufacturer. Cell/nucleic acid mixtures were combined with one of the 3 proprietary reagents: SE, SF, and SG, such that each reagent/cell/nucleic acid mixture occupies 1/3 of the 96-well electroporation plate. In each 1/3 of the plate, the cell/nucleic acid/reagent mixture is exposed to one of 31 different electric pulses, and 1 no-pulse control. In this way 96 conditions can be evaluated on a single plate, in which each well represents a different condition. Performing an optimization experiment in this way allows the evaluation of 96 different conditions; however since each well is represented only once, statistical reliability is absent. Repeating the optimization with the same 96-well conditions can be costly, and still not provide an appropriate biological replication in an individual experiment. We, therefore, used the initial optimization experiment as a 'screen' to pick the most promising reagent. Comprehensive data from this primary optimization can be observed in the supplementary file [see Additional file 1]. Following this, a secondary optimization was run using the best reagent in triplicate on a single 96-well plate. In this way, promising conditions were both repeated and biologically replicated.

**Figure 1:**
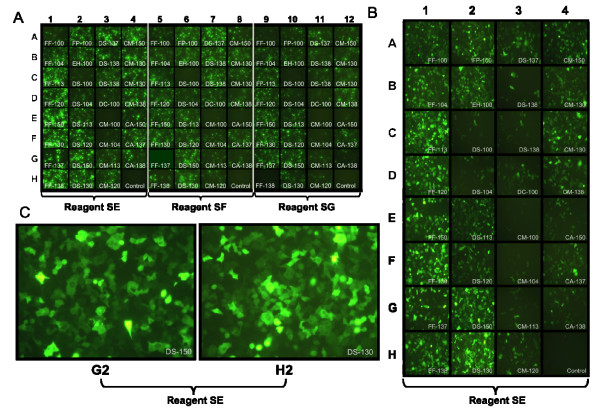
**Fluorescence microscopy of nucleofection optimizations**. (A) Microscopy images of the initial nucleofection optimization. Each well was subjected to a particular proprietary electroporation condition, designated by the serial number overlaid on each picture, and preceded by the number 96-(For example: Well B2 corresponds to 96-EH-100). Wells in columns 1–4 represent 32 different electroporation conditions, evaluating cells nucleofected in proprietary reagent SE. Columns 5–8 repeat the same 32 electroporation conditions in proprietary reagent SF, while columns 9–12 evaluate the 32 conditions in reagent SG. Wells H4, H8, and H12 are controls that contained the respective nucleofection reagents, but were not electroporated. (B) Microscopy of the secondary optimization containing SE only. Microscopy is only shown for 1/3 of the plate, representing each unique electroporation condition. Well H4 is the control well which was not electroporated. (C) Well G2 from initial optimization and H2 from SE optimization showing GFP throughout the cells.

To characterize the range of possible outcomes for the 96 nuclefection conditions, we monitored GFP levels and cell viability, via microscopy, plate reader, and trypan blue counting. Microscopy of the 96-well initial primary optimization (screen) is shown in Figure 1A. Most of the wells had some degree of successful nucleofection of the GFP plasmid shown by a homogeneous expression of green fluorescence. The electrical pulse patterns also show consistency among the 3 different reagents and their resulting GFP expression in each well. Bright-field microscopy was also performed on each well in the 96-well screen, with many wells exhibiting both good cell morphology and good GFP expression (data not shown). Fluorescence from GFP was measured using a plate reader on both lysed and non-lysed cells. Cell lysis was performed in order to release and homogenize GFP fluorescence throughout the well and mitigate any non-homogeneous cell coverage or instrument detection per well. Green fluorescence readings from both lysed and non-lysed cells were compared to determine any differences in outcome between methods and good correlation was found between the two methods (r = 0.95). Overall, fluorescence microscopy and plate reader signals ranged from completely absent to highly fluorescent and cell viability ranged from 72 to 100%. Total live cell number ranged from 6,000 to 193,000, with some wells showing massive cell loss following nucleofection.

As expected, an inverse relationship was observed between GFP fluorescence and cell viability. Therefore, we needed to determine the nucleofection conditions that can simultaneously provide moderate live cell number, high GFP fluorescence and nominal cell integrity as determined by microscopy. Based upon the fluorescence microscope images in Figure 1A and 1C and the analysis of a heat map containing all the results in a standardized form (data not shown), it was determined that the conditions used in well G2 (Reagent SE, program 96-DS:150) yielded cells with the best combination of results: the maximum GFP fluorescence, a total live cell count of 80,000 cells which falls above the median (68,500), nominal cell morphology, and high fluorescence under the microscope.

### Secondary Optimization

**Figure 2:**
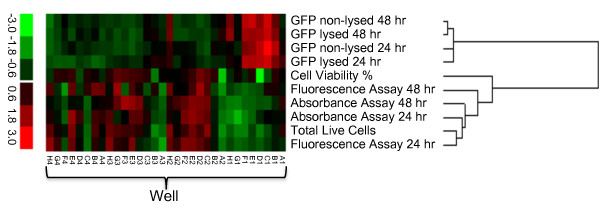
**Heat map of data from the secondary SE optimization**. Data has been standardized, with colors indicating high and low values. As seen on the scale, red indicates a high value relative to the mean of the individual data set, while green indicates a low value relative to the mean of the individual data set. Well H2 represents the best combination of GFP fluororescence, cell number, and cell viability.

In order to confirm the initial screening, observe any variation, and evaluate the most promising conditions, a second optimization was performed using the reagent with the best transfection characteristics determined from the screen. Based on the GFP microscopy and fluorescence and the live cell numbers from the initial optimization, reagent SE gave the best overall results of any reagent, and was therefore used in the secondary optimization. The results of the secondary SE optimization are shown in the microscopy images in Figure 1B, the Secondary Optimization data in the supplementary file [see Additional file 1], and the heat map in Figure 2 which allows data to be easily compared and the best wells/conditions to be determined. Similar results were observed, with fluorescence ranging from completely absent to highly fluorescent and cell viability ranging from 68% to 100%. Total live cell number ranged from 1,500 to 134,000, with some wells showing massive cell loss following nucleofection. The optimal condition determined from the secondary nucleofection is in well H2 (Figure 1C) which showed high GFP fluorescence, nominal morphology via microscopy, and a moderate live cell number of 73,000 which falls above the median (45,500). Interestingly, the optimal well determined here differed from that determined in the initial nucleofection, illustrating the utility of a second optimization.

In general, the variation among the three trials in each condition was large [see Additional file 1]. Even though conditions were averaged to increase reliability, it is worth noting that, in what appear to be identical replicates, moderate differences can be expected in nucleofection efficiencies, an important consideration in downstream experiments.

### Minimal Evaluation Assay Determination

To determine the simplest assays needed to find the optimal condition, we directly compared results for each assay. Comparing both the clustering hierarchy in the heat map (Figure 2), and the correlation values between redundant assays (Table 1), we determined that only a few measurements are needed to evaluate any given 96-well shuttle nucleofection experiment. The lysed vs. non-lysed GFP from both 24 and 48 hr correlate closely, which indicates that the GFP can be reliably measured on the 96-well plate without the addition of a lysis reagent. In addition, the total live cell number correlates well with both the absorbance cell viability assay and the fluorescence cell viability assay at 24 hr. The correlation is less clear at 48 hr, possibly due to different maximum limits of detection between the assays. In fact, for the secondary optimization, each individual assay agrees on the same optimal well condition (H2). This suggests that one need only measure non-lysed GFP fluorescence (using a plate reader) and cell viability by a simple assay (either the absorbance or fluorescence based) to evaluate the effects of a given condition/cell line for nucleofection.

### Delivery of siRNA and observable knock down of targeted genes

**Figure 3:**
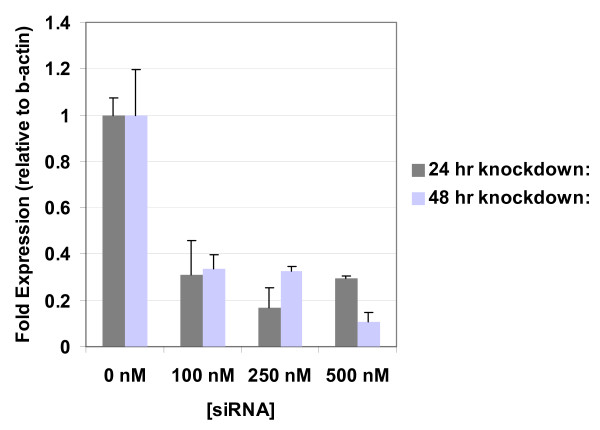
**QPCR Results**. QPCR of rel A knockdown in A-549s after 24 (grey) and 48 (blue) h.

Finally, to demonstrate efficient knockdown, we used one of the optimized conditions to deliver siRNA constructs using nucleofection with the aim of knocking down expression of human rel-a (NM_021975). The siRNA preparation consisted of a pooled sample of 4 sense, and 4 antisense sequences corresponding to 4 different regions of the target gene. The pooling of low concentrations of the 4 different sense/antisense pairs into 1 sample allows for a combinatorial targeting of the gene and limits off-target effects brought on by using higher concentrations of just a single pair. In addition, siRNAs are chemically modified to inhibit other off target affects, such as those caused by unfavourable RISC interaction of the seed strand, and anti-sense strand-related off-targeting induced by similar 3'UTR seeds [20,21]. Figure 3 shows the knockdown as measured by qPCR at 24 and 48 h for rel A over several concentrations delivered. In all cases, the transcripts were consistently knocked down to levels 70–95% lower than that detected in the controls.

## Discussion

Following an initial screening, we picked the SE reagent as the best choice to continue with a secondary optimization, although one could also pick combinations of the best wells from other reagents and evaluate them in triplicate under similar conditions. The use of a heat map to compare GFP and cell viability side by side is useful for determining the best conditions for nucleofection (Figure 2). For example, high GFP (and therefore high GFP/cell) would not necessarily be the best condition due to low total cell survival. The use of the heat map shows that well H2 yields the best combination of high GFP and viability. Conditions that yield large negative correlations between GFP fluorescence and cell number can also be compared and screened, and excessive cell deaths due to condition-related toxicity can be readily observed, such as cell death caused by lethal amounts of calcium-influx from electroporation-mediated holes in the membrane.

Certain assays are nominal for evaluating optimization, and do not require further refinement. For example, the linear, highly correlated relationship that exists between measurements of lysed and non-lysed GFP, as well as between trypan-blue counting and different cell viability assays. That is, one needs only to measure GFP fluorescence in intact cells, and perform a simple commercial 96-well cell viability assay to get reliable data. Therefore, the use of the screen plus secondary optimization, in conjunction with one of the fluorescence and cytotoxicity assays used here, can help determine the best balance of delivery and cell survival.

We have also used the delivery of siRNA pooled samples targeting rel A as a test platform to assay this optimized format for A-549 cells and shown the successful knockdown of rel A in a time- and concentration-dependent manner. The effective siRNA concentration range that we observe here is typical of standard concentrations used for single gene knockouts recommended by the manufacturer (250 nM-500 nM) as well as for genomic or pathway multi-gene knockdown screening (100 nM). Despite the success of delivery and the observable transcript phenotype, we did not optimize the nucleofection system here for siRNA delivery, but only for pmaxGFP plasmid delivery. Therefore, better conditions might exist to achieve a higher and more sustained knock down using this system.

## Conclusion

The introduction of the 96-well nucleofection shuttle system facilitates powerful gene delivery applications, allowing large numbers of conditions and replicates to be performed, and will find uses in high throughput screening, systematic knockdown studies, and even for ex vivo gene therapy applications. It is easy to use, attaining high transfection efficiencies and homogeneous intercellular distribution of the delivered nucleic acid within both the cytoplasm and the nuclear barrier. Therefore, siRNA delivery will also likely penetrate the nuclear envelope, leading to a more sustained knock-down. Optimization using this methodology can be carried out to determine the best conditions for each cell line so as to mitigate cell deaths and cell-proliferation inhibition, and to increase efficient transfection conditions. However, investigators should be aware of variations in individual replicates and take steps to mitigate their effects on outcomes, such as nucleic acid delivery and cell viability. In summary, we were able to optimize nucleofection conditions for A549 cells, define the minimal assays needed for the evaluation of 96-well shuttle results, and deliver an siRNA targeting complex through nucleofection in a 96-well format. The methods and results described here are widely applicable to those wanting to implement this technology for use in any cell line.

## Competing interests

The authors declare that they have no competing interests.

## Authors' contributions

CB and KR contributed equally to this manuscript. CB designed experiments and co-performed with KR all nucleofections, microscopy, cell counting, and cytotoxicity assays. CB also performed the qPCR, and drafted significant portions of the manuscript. KR co-performed all nucleofections, microscopy, cell counting, cytotoxicity assays, and drafted parts of the manuscript. KR also generated statistical correlation and heat map data. DT contributed intellectual direction, guidance and support, and editing of the manuscript.

## Supplementary Material

Additional file 1 **Comprehensive data from primary and secondary optimizations**. File containing raw data and standardized data produced in this study. The first sheet contains data from the primary optimization, and the second sheet contains data from the secondary optimization. Data includes live cell numbers, % viability of cells, non-lysed and lysed GFP fluorescence, absorbance assay results, cytotoxicity/fluorescent based assay results, and the standardized data. Click here for file
